# *Rickettsia felis* in Chile

**DOI:** 10.3201/eid1311.070782

**Published:** 2007-11

**Authors:** Marcelo B. Labruna, Maria Ogrzewalska, Jonas Moraes-Filho, Paulina Lepe, Jose Luis Gallegos, Javier López

**Affiliations:** * University of São Paulo, São Paulo, Brazil; † Alcantara Veterinary Clinics, Santiago, Chile

**Keywords:** Rickettsia felis, Ctenocephalides felis, fleas, cats, Chile, letter

**To the Editor:** Rickettsiosis due to *Rickettsia felis* is an emerging disease that has been reported worldwide (*1*). Fever, headache, myalgia, and macular rash have been attributed to *R. felis* infection in humans (*1*). In South America, *R. felis* infection in fleas (mostly *Ctenocephalides* spp.) has been reported only in Brazil, Peru, and Uruguay (*2**–**3*). Although a growing number of articles have reported that *R. felis* is transmitted by fleas, the acquisition mechanism of *R. felis* by vertebrates or uninfected fleas in nature remains unknown (*4*).

Cats experimentally exposed to *R. felis–*infected fleas have been shown to become seropositive (*5*). However, neither serologic nor molecular evidence of *R. felis* infection has been reported in cats under natural conditions, despite the fact that most *C. felis* fleas are infected by *R. felis* (*6**,**7*).

In November 2006, we investigated the presence of rickettsial DNA in 30 *C. felis* fleas randomly collected from 22 domestic cats privately owned and housed indoors in a single household in Santiago, Chile. To detect rickettsial DNA in each individual flea, PCRs were performed that targeted a 398-nt fragment of the rickettsial *gltA* gene and an 856-nt fragment of the rickettsial *ompB* gene (*7**,**8*).

A total of 21 individual fleas (70%) yielded expected PCR products for both *gltA* and *ompB* genes. PCR *gltA* products from the 21 fleas and *ompB* products from 5 fleas were subjected to DNA sequencing as described (*7*). The *gltA* partial sequences obtained from 21 fleas were identical, as were the *ompB* partial sequences from 5 fleas. These sequences were 100% identical to corresponding sequences in the *R. felis* genome (GenBank accession no. CP000053).

Blood serum samples were collected from the 22 cats and tested by indirect immunofluorescence assay (IFA) with crude antigens derived from 6 *Rickettsia* isolates from Brazil: *R. bellii*, *R. amblyommii*, *R. rhipicephali*, *R. rickettsii*, *R. parkeri*, and *R. felis* (*7*,*9*). Serum was considered to contain antibodies against rickettsiae if it displayed a reaction at 1:64 dilution. End-point titers against each *Rickettsia* species were determined by testing serial 2-fold serum dilutions. Reactive serum specimens were tested in 2 or 3 replications by 2 readers before the end-point titer was determined. Serum showing a *Rickettsia* species titer at least 4-fold higher than those observed for the other *Ricketttsia* species was considered homologous to the first *Rickettsia* species or to a very closely related genotype (*7**,**9*). In each slide, a nonreactive cat serum specimen (negative control) and a known reactive cat serum specimen (positive control) were tested at the 1:64 dilution (*7*).

IFA detected antibodies reactive with *R. felis* (titer >64) in 16 (72.7%) of 22 cats. Among those, 5 (22.7%) also reacted with *R. rhipicephali*, 4 (18.2%) with *R. bellii,* 3 (13.6%) with *R. parkeri,* 2 (9.1%) with *R. rickettsii,* and 1 (4.5%) with *R*. *amblyommii.* No serum reacted with any other *Rickettsia* species without reacting with *R. felis* (Table). Four cat serum specimens (cats 1, 3, 8, and 11) showed titers to *R. felis* at least 4-fold higher than those to any of the other 5 antigens. The antibody titers in these 4 animals were considered to have been stimulated by *R. felis* infection. For the remaining 12 seropositive cats, we could not discern whether *R. felis* had been the infection agent because the results displayed a single titer of 64 for *R. felis* or showed similar titers for other *Rickettsia* species.

**Table:** End-point titers of indirect immunofluorescence assay (IFA) for 6 *Rickettsia* species in cats from a household in Santiago, Chile*

Cat no.	IFA titers for *Rickettsia* antigens						PAIHR
	*R. felis*	*R. rhipicephali*	*R. bellii*	*R. parkeri*	*R. rickettsii*	*R. amblyommii*	
1	128	–	–	–	–	–	*R. felis*
2	256	256	128	–	–	–	
3	512	128	64	–	–	–	*R. felis*
5	64	–	–	–	–	–	
6	64	64	–	–	–	–	
7	64	–	–	–	–	–	
8	128	–	–	–	–	–	*R. felis*
9	64	–	–	–	–	–	
10	64	–	–	–	–	–	
11	128	–	–	–	–	–	*R. felis*
12	64	–	–	–	–	–	
14	128	–	64	128	–	128	
15	64	–	–	–	–	–	
19	64	–	–	–	–	–	
21	128	64	128	128	128	–	
22	64	64	–	64	64	–	

*PAIHR, possible antigen involved in a homologous reaction (serum showing for a *Rickettsia* species titer at least 4-fold higher than that observed for any other *Ricketttsia* species was considered homologous to the first *Rickettsia* species); –, nonreactive at titer >64.

We report 70% *R. felis*–infected fleas in this study on the basis of the concordant results of 2 PCR amplifications (*gltA* and *ompB*) and DNA sequencing. This infection rate is within the range (13.5%–90%) that has been reported for *R. felis* infecting *Ctenocephalides* fleas in Brazil and Uruguay (*2**,**3**,**7*). Sixteen (72.7%) cats contained *R. felis–*reactive antibodies; 4 of them showed titers to *R. felis* at least 4-fold higher than those to the other 5 rickettsial strains, findings that enabled us to technically conclude that these cats were exposed to *R. felis* or a closely related organism (*1**,**7**,**9*). Our finding of 70% *R. felis* infection in fleas infesting the cats indicates that cats acquired the infection through infected fleas. However, the mechanism of *R. felis* transmission by fleas is yet to be demonstrated under experimental conditions.

To our knowledge, the presence of *R. felis*, or a spotted fever group *Rickettsia* species, has not been reported in Chile. Recent investigations have provided clinical and serologic evidence of canine (*10*) and human (K. Abarca and J. Lopez, unpub. data) infection by spotted fever rickettsia in Chile, confirmed by IFA that used *R. conorii* commercial antigen. Since substantial serologic cross-reaction occurs between *R. conorii* and *R. felis* antigens (*1*), *R. felis* could be causing infection in dogs or humans in Chile.
